# *Setd5* is required in cardiopharyngeal mesoderm for heart development and its haploinsufficiency is associated with outflow tract defects in mouse

**DOI:** 10.1002/dvg.23421

**Published:** 2021-05-29

**Authors:** Michelle Yu‐Qing Cheung, Catherine Roberts, Peter Scambler, Athanasia Stathopoulou

**Affiliations:** ^1^ Developmental Biology and Cancer University College London Great Ormond Street Institute of Child Health 30 Guilford Street London WC1N 1EH United Kingdom; ^2^ Institute of Medical and Biomedical Education St. George's, University of London Cranmer Terrace London SW17 0RE United Kingdom

**Keywords:** birth defects, early development, heart, mesoderm

## Abstract

Congenital heart defects are a feature of several genetic haploinsufficiency syndromes, often involving transcriptional regulators. One property of haploinsufficient genes is their propensity for network interactions at the gene or protein level. In this article we took advantage of an online dataset of high throughput screening of mutations that are embryonic lethal in mice. Our aim was to identify new genes where the loss of function caused cardiovascular phenotypes resembling the 22q11.2 deletion syndrome models, that is, heterozygous and homozygous loss of *Tbx1*. One gene with a potentially haploinsufficient phenotype was identified, *Setd5*, thought to be involved in chromatin modification. We found murine *Setd5* haploinsufficiency to be associated with double outlet right ventricle and perimembranous ventricular septal defect, although no genetic interaction with *Tbx1* was detected. Conditional mutagenesis revealed that *Setd5* was required in cardiopharyngeal mesoderm for progression of the heart tube through the ballooning stage to create a four‐chambered heart.

## 1 INTRODUCTION

Syndromic congenital heart disease (CHD) encompasses several haploinsufficiency conditions. Haploinsufficient genes may be present at a “hub” of interacting networks (Huang, Lee, Marcotte, & Hurles, 2010), and phenotypic similarity has been used in human genetics to cluster syndromes based on the premise that these groupings inform biological relationships of the genes involved that is, the underlying genes may function within the same developmental “module” (Oti & Brunner, 2007). The candidate gene for 22q11.2 deletion syndrome (22q11DS) is *TBX1*, and is haploinsufficient in mice (Jerome & Papaioannou, 2001; Merscher et al., 2001). In mice, *Tbx1* heterozygotes present with hypo‐ or aplasia of the fourth PAA at embryonic day (E)10.5 which gives rise to great vessel defects such as interrupted aortic arch type B and aberrant right subclavian artery at later stages. Other cardiovascular defects include double outlet right ventricle (DORV) and ventricular septal defect (VSD) (Lindsay et al., 2001; Merscher et al., 2001).

An initiative entitled Deciphering the Mechanisms of Developmental Disorders (DMDD) aimed to take advantage of embryonic lethal mouse lines containing insertions that initially offer a gene‐trap‐like reporter allele with heterozygous or hypomorphic effect (https://dmdd.org.uk/; Mohun et al., 2013). Upon Flpe recombination, this converts to a conditional allele offering the potential for tissue‐specific studies of heart and brain development. Thus, the DMDD browser provides an online database of mutants where standard phenotype ontology terms can be used to search for genes with phenotypes related to known birth defect syndromes. The database contains high‐resolution episcopic microscopic (HREM) images of embryos at E9.5 and E14.5 allowing local rephenotyping. The database contained 209 genes analysed at E9.5 and E14.5. Querying the online browser with key phenotype ontology terms that define the *Tbx1* haploinsufficient phenotype such as “DORV” and “VSD” generated a list of genes that caused these defects when mutated. Out of the 209 total genes, a mutation in only a single gene caused DORV and VSD when heterozygous: *Setd5* (MGI allele: 4432631). We concluded *Setd5* was a strong candidate for causing haploinsufficient cardiac defects that are similar to those observed due to *Tbx1* haploinsufficiency. Moreover, there was a severe loss of function, lethal phenotype in *Setd5* nulls (https://dmdd.org.uk/).

*Setd5*, a ubiquitously expressed gene (Osipovich, Gangula, Vianna, & Magnuson, 2016), is thought to act as a histone modifier. Unlike other SET‐domain proteins, SETD5 appears to lack histone methyltransferase activity (Deliu et al., 2018; Osipovich et al., 2016). Immunoprecipitation and mass spectrometry studies indicate SETD5 associated with HDAC3, a protein complex containing histone‐deacetylating activity; however, there is conflicting evidence on whether SETD5 is required for HDAC3 recruitment (Deliu et al., 2018; Nakagawa et al., 2020; Osipovich et al., 2016). Clinical reports reveal the implications of *SETD5* haploinsufficiency in human intellectual disability, with some patients presenting with cardiac abnormalities (Fernandes et al., 2018; Grozeva et al., 2014; Szczałuba et al., 2016). Furthermore, CHD is present in up to 50% of patients with deleterious mutations in chromatin modification proteins underlying neurodevelopmental or psychiatric syndromes (Zaidi & Brueckner, 2017). Despite this, the role of *Setd5* in cardiac development has not been extensively investigated.

Osipovich et al showed that mice lacking *Setd5* (MGI allele: 5576778) die before E10.5 and were developmentally delayed; embryos were severely underdeveloped with many presenting with hemorrhaging compared to littermate heterozygous controls (Osipovich et al., 2016). Interestingly, unlike the heterozygous embryos presented in the DMDD browser, Osipovich et al reported heterozygous mice as viable and indistinguishable from the wild‐type embryos. Furthermore, the embryonic lethality of *Setd5* nulls by E10.5 limits the full assessment of the cardiac phenotype. The purpose of our study was, primarily, to more fully delineate the cardiac phenotype caused by the disruption of *Setd5* expression, and secondarily to explore whether there was any genetic interaction between *Setd5* and *Tbx1*. Currently, attempts to understand the *Setd5* function are focused on neuronal contexts; here we show the importance of this protein during heart development.

## 2 MATERIALS AND METHODS

### 2.1 Mouse strains, breeding, and genotyping

All animal husbandry, maintenance, and procedures were carried out in accordance to the UK Home Office regulations. All mice were maintained on the C57Bl/6 background.

The *Setd5* ^Fl/Fl^ line was derived from the targeted mouse line (Setd5tm1a[EUCOMM]Wtsi MGI:4432631) (Wellcome Trust Sanger Institute) and kindly donated by the Basson laboratory (KCL). *Setd5* heterozygotes were generated by crossing the floxed line with mice expressing *Actin*‐Cre (Lewandoski, Meyers, & Martin, 1997). F1 mice harboring the *Actin*‐Cre were backcrossed with wild‐type mice in order to generate mice without the *Actin*‐Cre for use in downstream experiments. Generation of double heterozygotes involved breeding *Setd5* ^+/−^ with *Tbx1* ^lacZ/+^ heterozygotes, described previously (Lindsay et al., 2001). To generate *Tbx1*‐cKOs, *Setd5* ^Fl/Fl^ mice were crossed with *Tbx1* ^Cre/+^ heterozygous mice, described previously (Huynh, Chen, Terrell, & Baldini, 2007). To delete *Setd5* in the cardiopharyngeal mesoderm, *Setd5* ^Fl/Fl^ mice were bred with mice expressing *Mesp1*‐Cre, described previously (Saga et al., 1999). Timed matings for embryo collection involved pairing sexually mature mice overnight and checking the presence of a copulation plug the following morning, which indicated 0.5 days post copulation. The PCR strategies for genotyping are available upon request.

### 2.2 Tissue processing, whole‐mount analysis, and histological analysis

Whole embryos were fixed in 4% paraformaldehyde overnight at 4°C and then stored in PBS until required. For histological analyzes, whole embryos or hearts were dehydrated in ethanol, and then processed in Histoclear and several washes in wax. Samples were embedded in paraffin and cut into 10 μm sections, air‐dried overnight and then rehydrated through a decreasing gradient of ethanol and stained with hemotoxylin and eosin. Whole‐mount embryo pictures were taken using the Zeiss Lumar V12 Stereoscope. Histological sections were imaged using the Zeiss Axioplan or the slide scanning facility at UCL IQPath. Stage‐matching was performed by assessment of the limb buds and pharyngeal arches (Boehm et al., 2011; Musy et al., 2018).

### 2.3 Calculation of double heterozygote expected frequencies

In the absence of a synergistic genetic interaction, the expected frequency of defects in a double heterozygote in which two genes of interest are haploinsufficient is calculated as follows:

(A + B[1 – A])*n.

A = percentage of defects observed in one single heterozygote. B = percentage of defects observed in alternate single heterozygote. n = total number of double heterozygotes collected.

Taking the example of VSDs presented in Table 1, A = 75% = 0.75; B = 31% = 0.31; n = 14.

(0.75 + 0.31[1–0.75])* 14 = 12.

**TABLE 1:** Summary of heart defects observed in WT, *Setd5* heterozygotes (*Setd5* ^+/−^), *Tbx1* heterozygotes (*Tbx1* ^lacZ/+^) and double heterozygotes (*Setd5* ^+/−^; *Tbx1* ^lacZ/+^) at E14.5

H&E analysis					
	WT(*n* = 8)	*Setd5* ^+/−^ (*n* = 12)	*Tbx1* ^lacZ/+^ (*n* = 13)	*Setd5* ^+/−^; *Tbx1* ^lacZ/+^ (*n* = 14)	
				Observed	Expected *n* number
CAT	0	0	0	0	0
Rotational defects	0	6 (50%)*	1 (8%)	8 (57%)	8
VSD	1 (13%)	9 (75%)*	4 (31%)	12 (86%)	12
Great vessel defects					
AbRS	0	0	1 (8%)	3 (21%)	1

*Note*: A Fisher's exact test was performed to determine whether the defects observed in *Setd5* ^+/−^ were statistically significant compared to wild‐type control embryos (*p < .05 compared with WT). The expected frequency of defects in double heterozygotes (*Setd5* ^+/−^; *Tbx1* ^lacZ/+^) was calculated as described in the methods. Rotational defects encompass both double outlet right ventricle and overriding aorta.

Abbreviations: AbRS, aberrant right subclavian artery; CAT, common arterial trunk; VSD, ventricular septal defect.

### 2.4 Statistical analysis

To determine whether genetically modified mice or embryos presented at Mendelian ratios, Chi‐squared analysis was performed. To determine the association of genotype with the frequency of defects observed, Fisher's exact test was performed. A *p* value less than .05 was taken to be significant.

## 3 RESULTS

### 3.1 *Setd5* haploinsufficiency leads to outflow tract rotational defects and VSDs

*Setd5* ^Fl/Fl^ mice were derived from the targeted mouse line (*Setd5* ^tm1a[EUCOMM]Wtsi^). The floxed line was then bred with *Tmem163* ^Tg(ACTB‐cre)2Mrt^ (*Actin*‐Cre) mice in order to generate *Setd5* heterozygotes (*Setd5* ^+/−^) for use in downstream embryo experiments. At E14.5, normal heart development results in the septation of the outflow tract (OFT) into the pulmonary trunk and aorta, with an intact interventricular septum dividing the left and right ventricles. A rotational defect, such as DORV or overriding aorta, occurs when the OFT fails to align with the two future ventricles at the looping stage. Correct looping of the heart tube is necessary to align the two definitive outflow vessels with their corresponding ventricular chambers (Christoffels et al., 2000; Moorman & Christoffels, 2003). If the OFT is misaligned such that an abnormal amount of aortic blood originates from the right ventricle, then this is known as an overriding aorta. If more than 50% of the aortic blood arises from the right ventricle, the result is DORV (Creazzo, Godt, Leatherbury, Conway, & Kirby, 1998).

**FIGURE 1:**
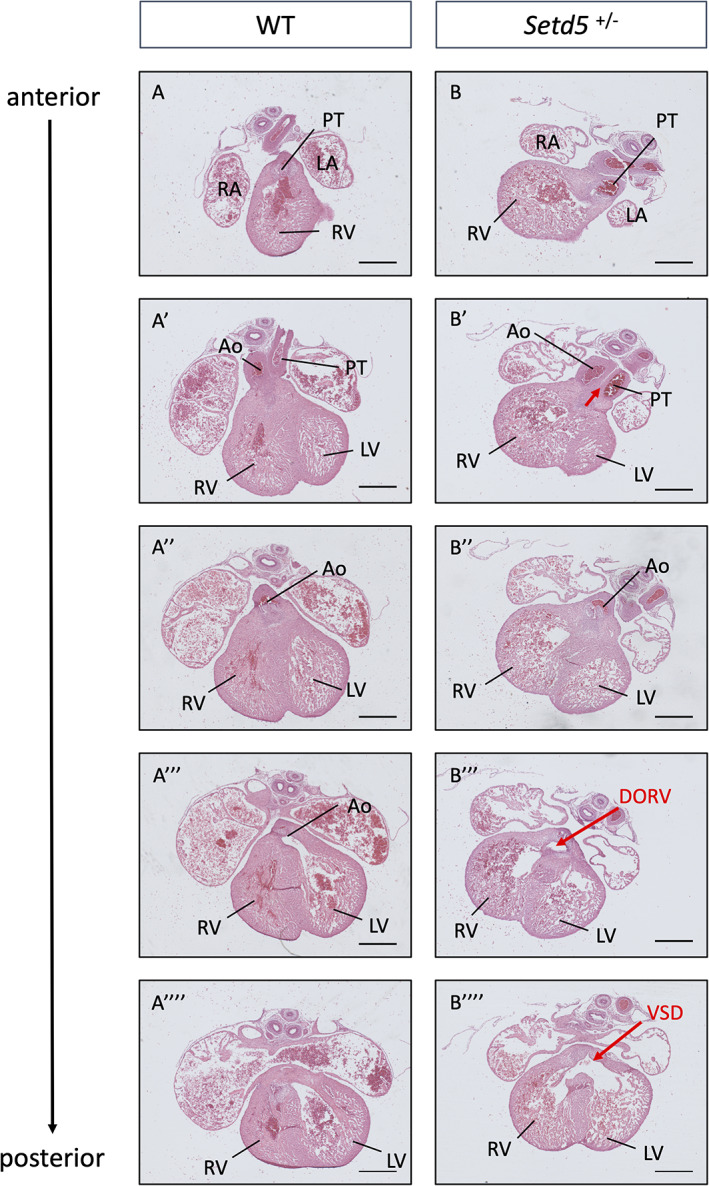
Rotational defects, including DORV was observed in 50% of *Setd5* heterozygotes (*Setd5* ^+/−^) and VSD were observed in 75% of *Setd5* ^+/−^ embryos at E14.5. In the WT heart, the pulmonary trunk and aorta open into separate ventricles: the right and left ventricle, respectively (a‑a‴). In *Setd5* ^+/−^ hearts, DORV was observed. In panel B′ the aorta and pulmonary trunk are juxtaposed, and in posterior sections b‴, the aorta eventually opens into the right ventricle; both the pulmonary trunk and aorta open into the right ventricle. A perimembranous VSD is observed in *Setd5* ^+/−^ (panel b″″). Ao, aorta; DORV, double outlet right ventricle; LA, left atrium; LV, left ventricle; PT, pulmonary trunk; RA, right atrium; RV, right ventricle; VSD, ventricular septal defects; WT, wild‐type. *Scale bars* = 400 μm

We found that wild‐type hearts displayed correctly OFT septation with the outflow vessels originating from separate ventricles (Figure 1a‑a‴). In contrast, despite correct OFT septation, 50% of *Setd5* heterozygotes presented with rotational defects which included DORV or overriding aorta (Figure 1b‑b‴) (*p* < .05, Fisher's exact test, Table 1). DORV is always accompanied by a VSD, since the fusion of the interventricular septum with the OFT cushions is necessary to complete ventricular septation. If the outflow vessels of the OFT are misaligned with the ventricular chambers, as in DORV, then this fusion cannot take place which leads to a perimembranous VSD (Anderson, 2003; Lin, Lin, Chen, Zhou, & Chang, 2012). Figure 1b″″ shows a perimembranous VSD, which was observed in 75% of *Setd5* heterozygotes (*p* < .05, Fisher's exact test, Table 1).

Therefore, in alignment with the DMDD browser, our results show that *Setd5* haploinsufficiency causes OFT rotation defects and VSDs.

### 3.2 No detectable genetic interaction between *Setd5* and *Tbx1*, a well‐established haploinsufficient gene

Because some of the phenotypes observed in *Setd5* heterozygotes overlap with those observed in *Tbx1* heterozygotes, the genetic interaction between *Setd5* and *Tbx1* was investigated by generating double heterozygotes, in which both genes of interest are haploinsufficient. *Setd5* heterozygotes were mated with *Tbx1* ^lacZ/+^ heterozygotes (*Tbx1* ^tm1Bld^) and embryos were harvested at E14.5. An exacerbation of defects, that is, an increased frequency of defects over that expected by additive effects, or an increase in severity of defects, would be observed in the double heterozygotes in the case of synergistic genetic interaction.

There was no difference in the number of embryos at E14.5 based on the genotypes (Table STable 1, Chi‐squared, *p* > .05). Gross morphological analysis during embryo dissection and internal assessment by histological methods revealed no exacerbation or increased frequency of defects in double heterozygotes, suggesting that there was no detectable synergistic genetic interaction between *Setd5* and *Tbx1* and that these two genes are not functionally interdependent (Table 1).

### 3.3 Conditional mutagenesis of *Setd5* reveals a role in cardiogenic mesoderm

**FIGURE 2:**
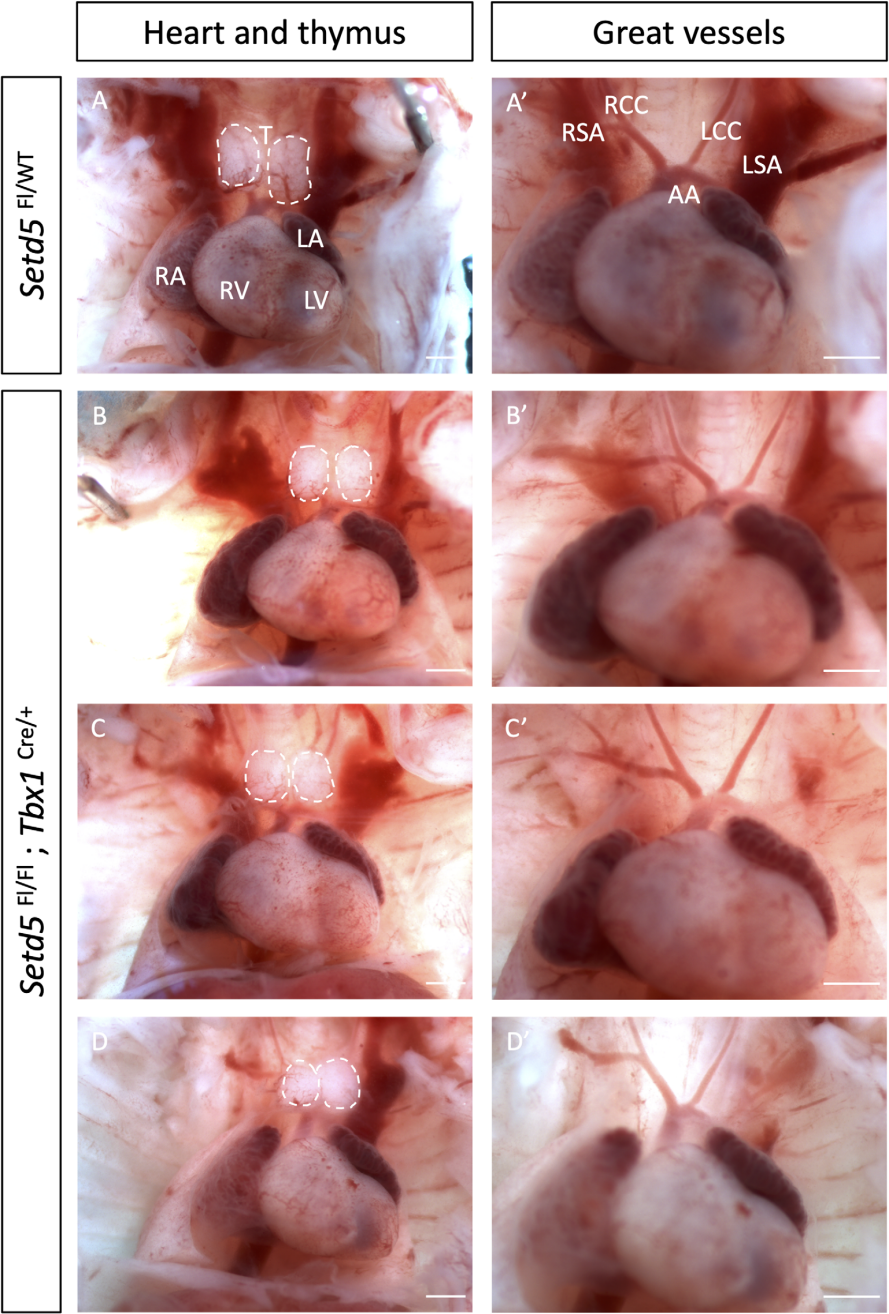
No obvious heart malformations were observed when *Setd5* was homozygously deleted using the *Tbx1‐Cre* at E15.5. No great vessel defects were observed across the genotypes (*n* = 19 conditional mutants analysed). AA, aortic arch; LA, left atria; LCC, left common carotid; LSA, left subclavian artery; LV, left ventricle; RA, right atria; RCC, right common carotid; RSA, right subclavian artery; RV, right ventricle; T, thymus. *Scale bars* = 1 mm

Our results show that the haploinsufficiency of *Setd5* led to rotational defects and VSD. These defects are reminiscent of the second heart field (SHF)‐specific defects seen in *Tbx1* mutants. To investigate whether *Setd5* has a specific role in the SHF, and/or pharyngeal epithelia, *Setd5* was deleted using a *Tbx1*‐Cre driver known to be active in these tissues (Huynh et al., 2007). Figure 2 presents the gross morphology of control and several *Tbx1* conditional knockout (*Tbx1*‐cKO) hearts at E15.5. Minor abnormalities such as retroesophageal right subclavian artery were observed (one in both *Tbx1* conditional heterozygote and knockout); this was likely due to the effect of the Cre knock‐in to the *Tbx1* locus. Similarly, assessment of internal heart morphology revealed no obvious differences between the genotypes (Figure SFigure 1). Therefore, we concluded *Setd5* likely exerts its role earlier during cardiac development, or in other lineages, or both.

**FIGURE 3:**
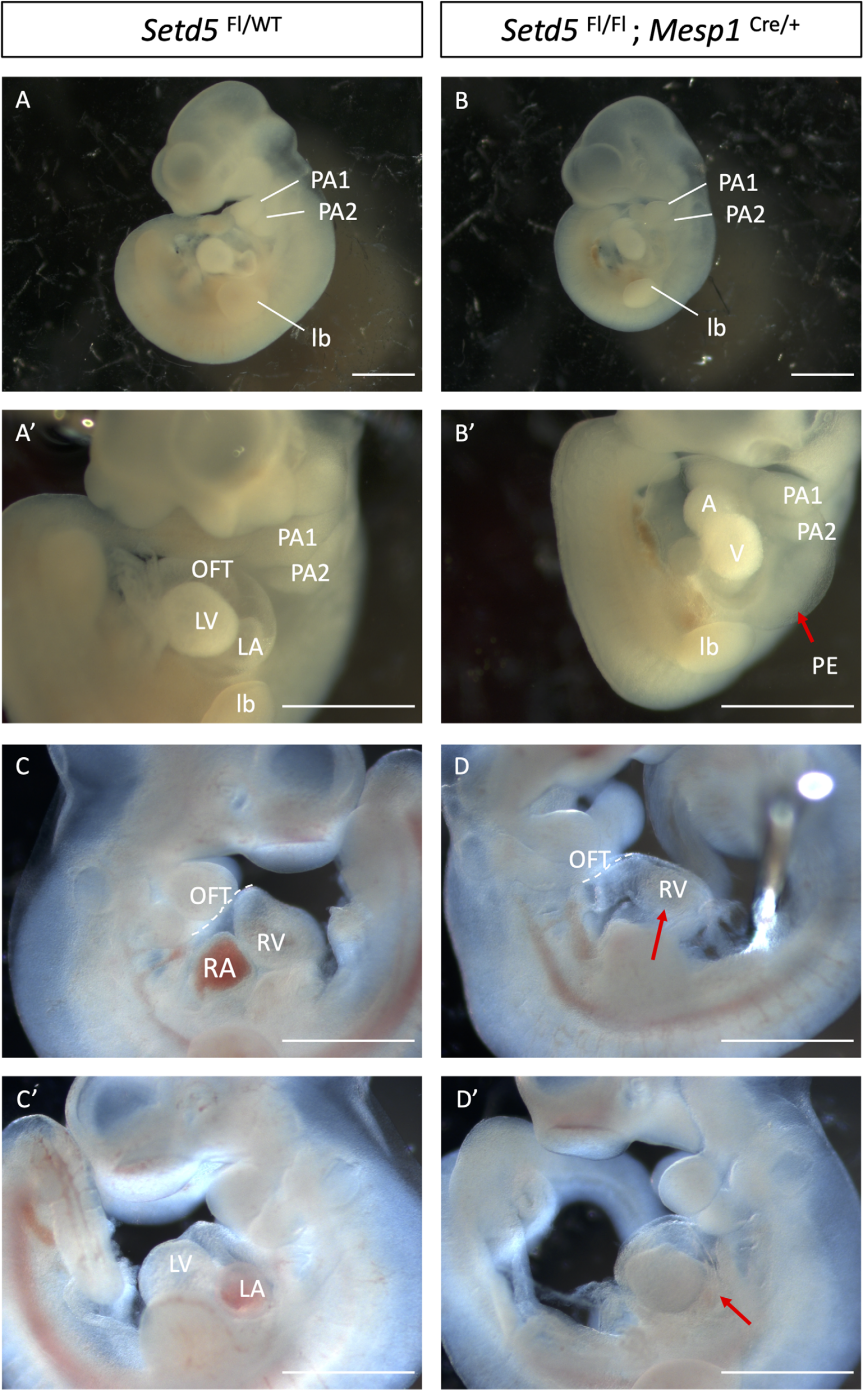
*Mesp1*‐cKO (*Setd5* ^Fl/Fl^; *Mesp1* ^Cre/+^) embryos displayed abnormal cardiac chamber ballooning and pericardial effusion at E10.5. At E10.5, *Mesp1*‐cKO embryos presented with abnormal cardiac morphogenesis in 90% of the cases with 25% of these exhibiting a dumbbell shaped heart: a single atria and single ventricle (b,b′) compared with control embryos (*Setd5* ^Fl/Fl^ or *Setd5* ^Fl/WT^). Pericardial effusion was observed in 70% of *Mesp1*‐cKO embryos (red arrow, b′). (d,d′) show a *Mesp1*‐cKO embryo with a short outflow tract, right ventricular hypoplasia, and abnormal atrial ballooning (red arrow). A, atrium; LA, left atrium; LV, left ventricle; lb, limb bud; OFT, outflow tract; PE, pericardial effusion; PAA, pharyngeal arch artery. *Scale bars* = 1 mm

**FIGURE 4:**
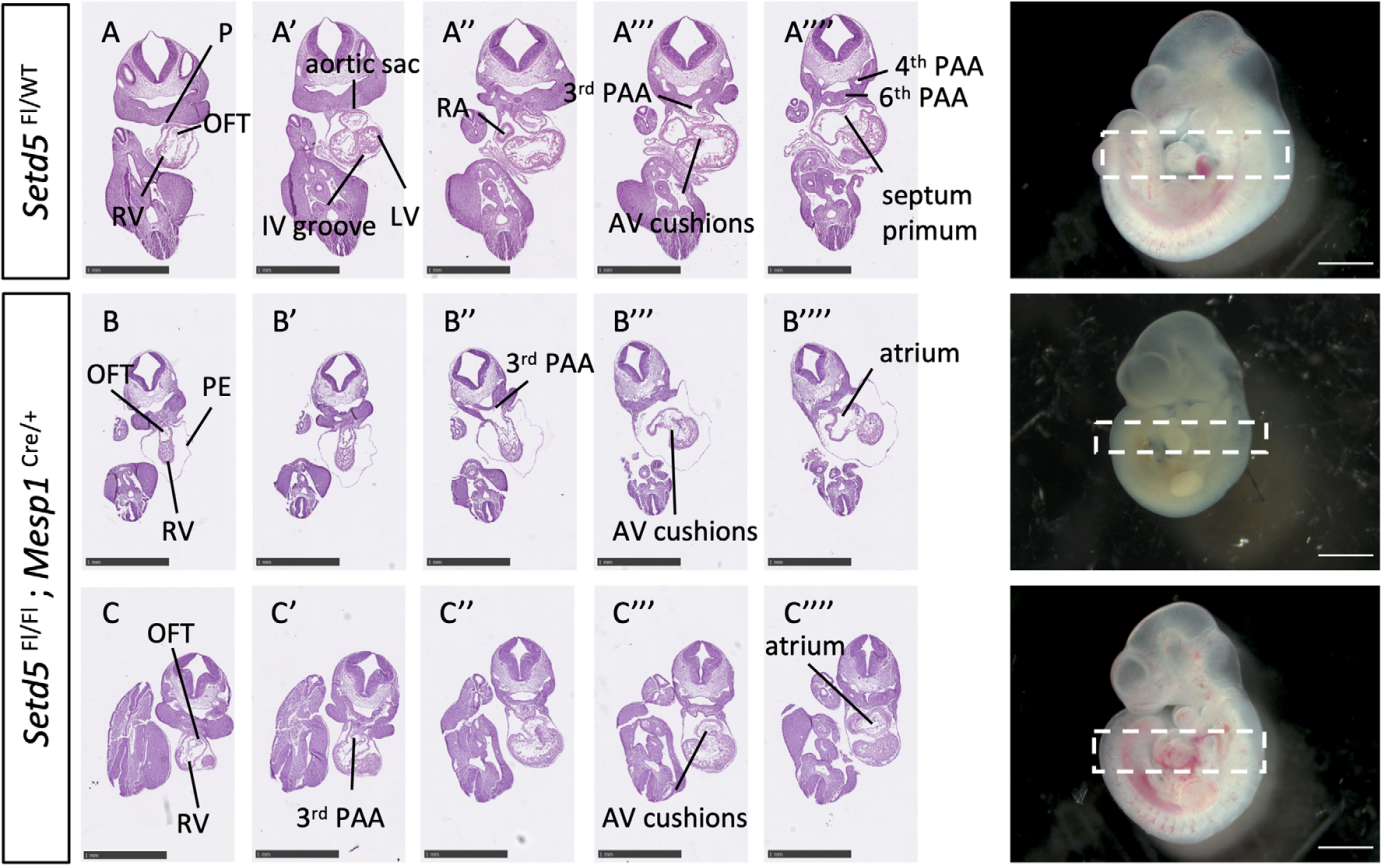
Short OFT and abnormal chamber morphology in *Setd5* ^Fl/Fl^; *Mesp1* ^Cre/+^ embryos at E10.5. (A‑A″″) Transverse sections of whole E10.5 *Setd5* ^Fl/WT^ control embryo stained with H&E. The IV groove is observed in panel A'. The emergence of the septum primum (A″″) occurs during atrial septation. (B‑B″″) Transverse sections of whole E10.5 *Setd5* ^Fl/Fl^; *Mesp1* ^Cre/+^ (cKO) embryo with dumbbell‐shaped heart. Short OFT is depicted by the fewer number of sections between the point at which the OFT emerged and when it fed into the 3rd PAA. The atria are poorly developed with a thickened wall (B””). (C‑C””) Transverse sections of whole E10.5 *Setd5* ^Fl/Fl^; *Mesp1* ^Cre/+^ (cKO) embryo with a slightly milder phenotype than in B. Embryo presents with a short OFT and no evidence of the interventricular groove, and thickened atrial wall. Sections presented are from top to bottom of the dashed box in whole embryo photograph. AV, atrioventricular; IV, interventricular; LV, left ventricle; OFT, outflow tract; PE, pericardial effusion; P, pericardium; PAA, pharyngeal arch artery; RA, right atrium; RV, right ventricle. *Scale bars* = 1 mm

Indeed, the DMDD browser revealed an embryonic lethality of homozygous *Setd5* mutation (no surviving embryos at E14.5), and Osipovich et al., showed that mice constitutively null for *Setd5* die before E10.5, and are severely underdeveloped. Hearts at E9.5 were swollen with a single ventricular chamber (Osipovich et al., 2016). The embryonic lethality of *Setd5* constitutive null embryos by E10.5 limits analysis of the importance of *Setd5* in cardiac development. Lineage tracing experiments show that the majority of cardiac cells are derived from *Mesp1*‐expressing progenitor cells (Saga et al., 1999). Therefore, we used the *Mesp1*‐Cre driver to delete *Setd5* in mesoderm encompassing both the first heart field (FHF) and SHF, as well as the pharyngeal mesoderm through which the (non‐*Mesp1*‐expressing) neural crest migrates. Homozygous deletion in this cardiopharyngeal mesoderm lineage led to embryonic lethality before E12.5. At E10.5, *Mesp1*‐cKO embryos were harvested at the expected Mendelian ratio (Table S2) but exhibited varying degrees of abnormal cardiac morphogenesis compared to stage‐matched control embryos (*p* < .05, Table 2). 90% of *Mesp1*‐cKOs displayed abnormal cardiac chamber ballooning, with 25% of these exhibiting a dumbbell‐shaped heart (Figure 3b), while others displayed right ventricular hypoplasia and abnormal atrial ballooning (*p* < .05, Figure 3d). We assessed the internal heart morphology by histological techniques and found that 100% of *Mesp1*‐cKO embryos presented with a short OFT, indistinct ventricular chambers, and poorly developed atria, revealing the importance of *Setd5* in OFT elongation and cardiac chamber ballooning (Figure 4). These defects were accompanied by a statistically significant growth delay, determined by the crown‐to‐rump length (*p* < .05, Figure SFigure 2). The OFT length was further assessed by quantifying the number of paraffin sections containing the OFT and expressing this as a ratio to the crown‐to‐rump length, confirming the shorter OFT in *Mesp1*‐cKO compared to control embryos (Figure SFigure 3). Morphological analysis of *Mesp1*‐Cre conditional heterozygotes (*n* = 6) did not reveal any OFT phenotype, and histological analysis revealed only a single embryo as having poorly developed cardiac chambers.

**TABLE 2:** Summary of defects observed in *Setd5* ^Fl/Fl^, *Setd5* ^Fl/WT^, *Setd5* ^Fl/WT^; *Mesp1* ^Cre/+^, and *Setd5* ^Fl/Fl^; *Mesp1* ^Cre/+^ embryos at E10.5

Abnormal external morphology				
	*Setd5* ^Fl/Fl^(*n* = 17)	*Setd5* ^Fl/WT^ (*n* = 23)	*Setd5* ^Fl/WT^ *; Mesp1* ^Cre/+^ (*n* = 17)	*Setd5* ^Fl/Fl^ *; Mesp1* ^Cre/+^ (*n* = 19)
Hemorrhage	0	0	0	3 (16%)
Pericardial effusion*	0	3 (13%)	0	14 (74%)
Abnormal cardiac chamber ballooning*	0	0	0	18 (95%)
**H&E analysis**				
	***Setd5* ^Fl/Fl^ (*n* = 2)**	***Setd5* ^Fl/WT^ (*n* = 4)**	***Setd5* ^Fl/WT^ *; Mesp1* ^Cre/+^ (*n* = 6)**	***Setd5* ^Fl/Fl^ *; Mesp1* ^Cre/+^ (*n* = 6)**
Short OFT*	0	0	0	6 (100%)
Abnormal ventricular ballooning*	0	0	1 (17%)	6 (100%)
Abnormal atrial ballooning*	0	0	0	6 (100%)

*Note*: The upper part of the table describes the defects observed during the dissection stage. The abnormal external cardiac morphology was further delineated by sectioning and H&E analysis. Fisher's exact test was used to determine the association between the genotype and the frequency of any defects observed.

* *p* < .05.

## 4 DISCUSSION

Our results indicate that haploinsufficiency of *Setd5*, a gene implicated in human intellectual disability (Fernandes et al., 2018; Grozeva et al., 2014; Szczałuba et al., 2016) with reported histone‐modifying activity (Deliu et al., 2018; Nakagawa et al., 2020; Osipovich et al., 2016), leads to cardiac defects in mice. It is worth noting that only one *Mesp1*‐Cre conditional heterozygote presented with poorly developed cardiac chambers. The remaining embryos exhibited normal cardiac morphology, highlighting the fact that deleting one allele of *Setd5* in the *Mesp1* lineage does not fully recapitulate the global heterozygotes and that haploinsufficiency of *Setd5* requires constitutive heterozygosity. Since haploinsufficient genes exist in hubs or networks and are more likely to interact with other haploinsufficient genes (Huang et al., 2010), and because haploinsufficiency of *Setd5*, and of *Tbx1* lead to similar cardiac defects, one of our initial aims were to investigate their genetic interaction. However, our findings did not reveal a detectable synergistic genetic interaction, suggesting that *Setd5* and *Tbx1* are not functionally interdependent. Nevertheless, homozygous deletion of *Setd5* in the cardiopharyngeal mesoderm revealed a novel role of *Setd5* in cardiac development: its importance in OFT elongation and cardiac chamber ballooning.

In humans, loss of function mutations in *SETD5* lead to moderate or severe intellectual disability (Fernandes et al., 2018; Grozeva et al., 2014; Powis et al., 2018; Rawlins, Stals, Eason, & Turnpenny, 2017; Szczałuba et al., 2016). It is also one of three genes within the candidate region of 3p25 microdeletion syndrome which is characterized by developmental delay, intellectual disability, low birth weight, microcephaly and craniofacial abnormalities, and some observations of CHD (Grozeva et al., 2014; Kuechler et al., 2015; Szczałuba et al., 2016). Mutations of *SETD5* have been reported in other conditions. For instance, one deletion and two mutations were described in patients clinically diagnosed with KBG syndrome without a mutation in *ANKRD11*, which is usually observed in these cases (OMIM 148050). One such patient presented with mitral stenosis (Crippa et al., 2020). Interestingly, ANKRD11 recruits HDACs and is mutated in autism (Gallagher et al., 2015). Recently, *SETD5* has been implicated in moyamoya angiopathy, which is the occlusion of large cerebral arteries leading to childhood stroke (Pinard et al., 2020). Our results reveal that *Setd5*, a gene implicated in neurodevelopmental disorders, is also important in cardiac development, and may provide some explanation to the clinical observations of CHD when *SETD5* is haploinsufficient in humans. Taken together, these data suggest considerable pleiotropy of *SETD5* function.

The genetic underpinnings of CHD remain unknown in over 50% of cases, highlighting the importance of identifying novel genes that may be involved in orchestrating cardiac development. Furthermore, genome sequencing CHD studies present an overrepresentation of genes involved in neurodevelopmental disorder (Sigmon, Kelleman, Susi, Nylund, & Oster, 2019; Watkins et al., 2019). As such, screening for mutations in *SETD5* during genetic testing could potentially provide some knowledge to families to better understand if there could be recurrent risks of CHD to other family members, or provide information for neurodevelopmental outcome.

Thus, our results provide the basis for further exploration of *Setd5* in cardiac development. It will be interesting to investigate how this histone modifier plays a role in orchestrating cardiac development in conjunction with other known cardiac‐relevant genes. *Setd5* can now be placed within a growing number of genes encoding chromatin modifiers whose alleles are associated with both neurodevelopmental defects on the one hand, and heart dysmorphogenesis on the other.

## Supporting information


**Supplementary Figure 1** **Transverse sections of hearts of *Tbx1*‐cKO (*Setd5*** ^**Fl/Fl**^ **; *Tbx1*** ^**Cre/+**^ **) at E15.5.**
Panels A – A" show correct outflow tract (OFT) septation across all genotypes. The atrial septum (B – B″) and AV valves (C – C″) were intact across all genotypes
Abbreviations: aorta (Ao), left atrium (LA), left ventricle (LV), pulmonary trunk (PT), right atrium (RA), right ventricle (RV). Scale bars = 200 μm.

**Supplementary Figure 2** **Box plot showing the crown‐to‐rump length in mm at E10.5.**
*Setd5* ^Fl/Fl^; *Mesp1* ^Cre/+^ cKO embryos are significantly smaller than control embryos (*Setd5* ^Fl/Fl^ or *Setd5* ^Fl/WT^, *p* < .05, one‐way ANOVA). Error bars present the standard error of mean.

**Supplementary Figure 3** **Graph showing the OFT length of control embryos (*Setd5*** ^**Fl/Fl**^ **or Setd5** ^**Fl/WT**^ **) and cKO embryos (*Setd5*** ^**Fl/Fl**^ **; *Mesp1*** ^**Cre/+**^ **)**
The OFT length is presented as a ratio of the number of H&E paraffin sections containing the OFT, to the crown‐to‐rump length. An unpaired student's *t* test showed that cKO embryos had a shorter OFT than control embryos (*p < .05). Error bars present the standard error of mean.

**Supplementary Table 1** **Observed and expected number of embryos at E14.5 resulting *Setd5*** ^**+/−**^ **x *Tbx1*** ^**lacZ/+**^ **cross.**
Expected numbers are based on Mendelian ratios, rounded to the nearest whole number. There was no statistical difference in the number of E14.5 embryos across the genotypes, based on Chi‐squared analysis, *p* > .05.
**Supplementary Table 2: Observed and expected number of embryos at E10.5 resulting *Setd5*** ^**Fl/Fl**^ **x *Setd5*** ^**Fl/WT**^ **; *Mesp1*** ^**Cre/+**^ **cross.**
Expected numbers are based on Mendelian ratios, rounded to the nearest whole number. There was no statistical difference in the number of E10.5 embryos across the genotypes, based on Chi‐squared analysis, *p* > .05.

## Data Availability

The data that support the findings of this study are available from the corresponding author upon reasonable request.

## References

[dvg23421-bib-0001] Anderson, R. H. (2003). Development of the heart: (3) formation of the ventricular outflow tracts, arterial valves, and intrapericardial arterial trunks. Heart, 89(9), 1110–1118. 10.1136/heart.89.9.1110 12923046 PMC1767864

[dvg23421-bib-0002] Boehm, B. , Rautschka, M. , Quintana, L. , Raspopovic, J. , Jan, Ž. , & Sharpe, J. (2011). A landmark‐free morphometric staging system for the mouse limb bud. Development, 138(6), 1227–1234. 10.1242/dev.057547 21307091 PMC3042875

[dvg23421-bib-0003] Christoffels, V. M. , Habets, P. E. M. H. , Franco, D. , Campione, M. , de Jong, F. , Lamers, W. H. , … Moorman, A. F. M. (2000). Chamber formation and morphogenesis in the developing mammalian heart. Developmental Biology, 223(2), 266–278. 10.1006/dbio.2000.9753 10882515

[dvg23421-bib-0004] Creazzo, T. L. , Godt, R. E. , Leatherbury, L. , Conway, S. J. , & Kirby, M. L. (1998). Role of cardiac neural crest cells in cardiovascular development. Annual Review of Physiology, 60(1), 267–286. 10.1146/annurev.physiol.60.1.267 9558464

[dvg23421-bib-0005] Crippa, M. , Bestetti, I. , Maitz, S. , Weiss, K. , Spano, A. , Masciadri, M. , … Finelli, P. (2020). SETD5 gene Haploinsufficiency in three patients with suspected KBG syndrome. Frontiers in Neurology, 11(July), 1–9. 10.3389/fneur.2020.00631 32793091 PMC7393934

[dvg23421-bib-0006] Deliu, E. , Arecco, N. , Morandell, J. , Dotter, C. P. , Contreras, X. , Girardot, C. , … Novarino, G. (2018). Haploinsufficiency of the intellectual disability gene SETD5 disturbs developmental gene expression and cognition. Nature Neuroscience, 21(12), 1717–1727. 10.1038/s41593-018-0266-2 30455454

[dvg23421-bib-0007] DMDD Deciphering the Mechanisms of Developmental Disorders. (2020). Retrieved from https://dmdd.org.uk/

[dvg23421-bib-0008] Fernandes, I. R. , Cruz, A. C. P. , Ferrasa, A. , Phan, D. , Herai, R. H. , & Muotri, A. R. (2018). Genetic variations on SETD5 underlying autistic conditions. Developmental Neurobiology, 78(5), 500–518. 10.1002/dneu.22584 29484850

[dvg23421-bib-0009] Gallagher, D. , Voronova, A. , Zander, M. A. , Cancino, G. I. , Bramall, A. , Krause, M. P. , … Miller, F. D. (2015). Ankrd11 is a chromatin regulator involved in autism that is essential for neural development. Developmental Cell, 32(1), 31–42. 10.1016/j.devcel.2014.11.031 25556659

[dvg23421-bib-0010] Grozeva, D. , Carss, K. , Spasic‐Boskovic, O. , Parker, M. J. , Archer, H. , Firth, H. V. , … Raymond, F. L. (2014). De novo loss‐of‐function mutations in SETD5, encoding a methyltransferase in a 3p25 microdeletion syndrome critical region, cause intellectual disability. The American Journal of Human Genetics, 94(4), 618–624. 10.1016/j.ajhg.2014.03.006 24680889 PMC3980521

[dvg23421-bib-0011] Huang, N. , Lee, I. , Marcotte, E. M. , & Hurles, M. E. (2010). Characterising and predicting haploinsufficiency in the human genome. PLoS Genetics, 6(10), 1–11. 10.1371/journal.pgen.1001154 PMC2954820 20976243

[dvg23421-bib-0012] Huynh, T. , Chen, L. , Terrell, P. , & Baldini, A. (2007). A fate map of Tbx1 expressing cells reveals heterogeneity in the second cardiac field. Genesis, 45(7), 470–475. 10.1002/dvg.20317 17610275

[dvg23421-bib-0013] Jerome, L. A. , & Papaioannou, V. E. (2001). DiGeorge syndrome phenotype in mice mutant for the T‐box gene, Tbx1. Nature Genetics, 27(3), 286–291. 10.1038/85845 11242110

[dvg23421-bib-0014] Kuechler, A. , Zink, A. M. , Wieland, T. , Lüdecke, H. J. , Cremer, K. , Salviati, L. , … Engels, H. (2015). Loss‐of‐function variants of SETD5 cause intellectual disability and the core phenotype of microdeletion 3p25.3 syndrome. European Journal of Human Genetics, 23(6), 753–760. 10.1038/ejhg.2014.165 25138099 PMC4795044

[dvg23421-bib-0032] Lewandoski, M. , Meyers, E. N. , & Martin, G. R. (1997). Analysis of Fgf8 gene function in vertebrate development. Cold Spring Harbor Symposia on Quantitative Biology, 62(1), 159–168. 10.1101/SQB.1997.062.01.021 9598348

[dvg23421-bib-0015] Lin, C.‐J. , Lin, C.‐Y. , Chen, C.‐H. , Zhou, B. , & Chang, C.‐P. (2012). Partitioning the heart: Mechanisms of cardiac septation and valve development. Development, 139(18), 3277–3299. 10.1242/dev.063495 22912411 PMC3424040

[dvg23421-bib-0016] Lindsay, E. A. , Vitelli, F. , Su, H. , Morishima, M. , Huynh, T. , Pramparo, T. , … Baldini, A. (2001). Tbx1 haploinsufficieny in the DiGeorge syndrome region causes aortic arch defects in mice. Nature, 410(6824), 97–101. 10.1038/35065105 11242049

[dvg23421-bib-0017] Merscher, S. , Funke, B. , Epstein, J. A. , Heyer, J. , Puech, A. , Lu, M. M. , … Kucherlapati, R. (2001). TBX1 is responsible for cardiovascular defects in velo‐cardio‐facial/DiGeorge syndrome. Cell, 104(4), 619–629. 10.1016/S0092-8674 11239417

[dvg23421-bib-0018] Mohun, T. , Adams, D. J. , Baldock, R. , Bhattacharya, S. , Copp, A. J. , Hemberger, M. , … Weninger, W. (2013). Deciphering the mechanisms of developmental disorders (DMDD): A new programme for phenotyping embryonic lethal mice. Disease Models & Mechanisms, 6(3), 562–566. 10.1242/dmm.011957 23519034 PMC3634640

[dvg23421-bib-0019] Moorman, A. F. M. , & Christoffels, V. M. (2003). Cardiac chamber formation: Development, genes, and evolution. Physiological Reviews, 83(4), 1223–1267. 10.1152/physrev.00006.2003 14506305

[dvg23421-bib-0020] Musy, M. , Flaherty, K. , Raspopovic, J. , Robert‐Moreno, A. , Richtsmeier, J. T. , & Sharpe, J. (2018). A quantitative method for staging mouse embryos based on limb morphometry. Development (Cambridge), 145(7), 1–7. 10.1242/dev.154856 PMC5963863 29540505

[dvg23421-bib-0021] Nakagawa, T. , Hattori, S. , Nobuta, R. , Kimura, R. , Nakagawa, M. , Matsumoto, M. , … Nakayama, K. (2020). The autism‐related protein SETD5 controls neural cell proliferation through epigenetic regulation of rDNA expression. IScience, 23(4), 101030. 10.1016/j.isci.2020.101030 32299058 PMC7160574

[dvg23421-bib-0022] Osipovich, A. B. , Gangula, R. , Vianna, P. G. , & Magnuson, M. A. (2016). Setd5 is essential for mammalian development and the co‐transcriptional regulation of histone acetylation. Development, 143(24), 4595–4607. 10.1242/dev.141465 27864380 PMC5201031

[dvg23421-bib-0023] Oti, M. , & Brunner, H. G. (2007). The modular nature of genetic diseases. Clinical Genetics, 71(1), 1–11. 10.1111/j.1399-0004.2006.00708.x 17204041

[dvg23421-bib-0024] Pinard, A. , Guey, S. , Guo, D. , Cecchi, A. C. , Sharrief, A. Z. , Bergametti, F. , … Tournier‐lasserve, E. (2020). The pleiotropy associated with de novo variants in CHD4, CNOT3, and SETD5 extends to moyamoya angiopathy. Genetics in Medicine, 22(2), 427–431. 10.1038/s41436-019-0639-2 31474762 PMC7673309

[dvg23421-bib-0025] Powis, Z. , Farwell Hagman, K. D. , Mroske, C. , McWalter, K. , Cohen, J. S. , Colombo, R. , … Tang, S. (2018). Expansion and further delineation of the SETD5 phenotype leading to global developmental delay, variable dysmorphic features, and reduced penetrance. Clinical Genetics, 93(4), 752–761. 10.1111/cge.13132 28881385

[dvg23421-bib-0026] Rawlins, L. E. , Stals, K. L. , Eason, J. D. , & Turnpenny, P. D. (2017). De novo SETD5 nonsense mutation associated with diaphragmatic hernia and severe cerebral cortical dysplasia. Clinical Dysmorphology, 26(2), 95–97. 10.1097/MCD.0000000000000144 28263952

[dvg23421-bib-0027] Saga, Y. , Miyagawa‐Tomita, S. , Takagi, A. , Kitajima, S. , Miyazaki, J. , & Inoue, T. (1999). MesP1 is expressed in the heart precursor cells and required for the formation of a single heart tube. Development, 126(15), 3437–3447.Retrieved from. http://www.ncbi.nlm.nih.gov/pubmed/10393122 10393122 10.1242/dev.126.15.3437

[dvg23421-bib-0028] Sigmon, E. R. , Kelleman, M. , Susi, A. , Nylund, C. M. , & Oster, M. E. (2019). Congenital heart disease and autism: A case‐control study. Pediatrics, 144(5), 1–8. 10.1542/peds.2018-4114 31601611

[dvg23421-bib-0029] Szczałuba, K. , Brzezinska, M. , Kot, J. , Rydzanicz, M. , Walczak, A. , Stawiński, P. , … Płoski, R. (2016). SETD5 loss‐of‐function mutation as a likely cause of a familial syndromic intellectual disability with variable phenotypic expression. American Journal of Medical Genetics, Part A, 170(9), 2322–2327. 10.1002/ajmg.a.37832 27375234

[dvg23421-bib-0030] Watkins, W. S. , Hernandez, E. J. , Wesolowski, S. , Bisgrove, B. W. , Sunderland, R. T. , Lin, E. , … Tristani‐Firouzi, M. (2019). De novo and recessive forms of congenital heart disease have distinct genetic and phenotypic landscapes. Nature Communications, 10(1), 1–12. 10.1038/s41467-019-12582-y PMC6797711 31624253

[dvg23421-bib-0031] Zaidi, S. , & Brueckner, M. (2017). Genetics and genomics of congenital heart disease. Circulation Research, 120(6), 923–940. 10.1161/CIRCRESAHA.116.309140 28302740 PMC5557504

