# Supplementary figures and images for: Setd5 is required in cardiopharyngeal mesoderm for heart development and its haploinsufficiency is associated with outflow tract defects in mouse
Source: Genesis. 2021 May 29;59(7-8):e23421. doi: 10.1002/dvg.23421 (PMC8564859; doi:10.1002/dvg.23421)

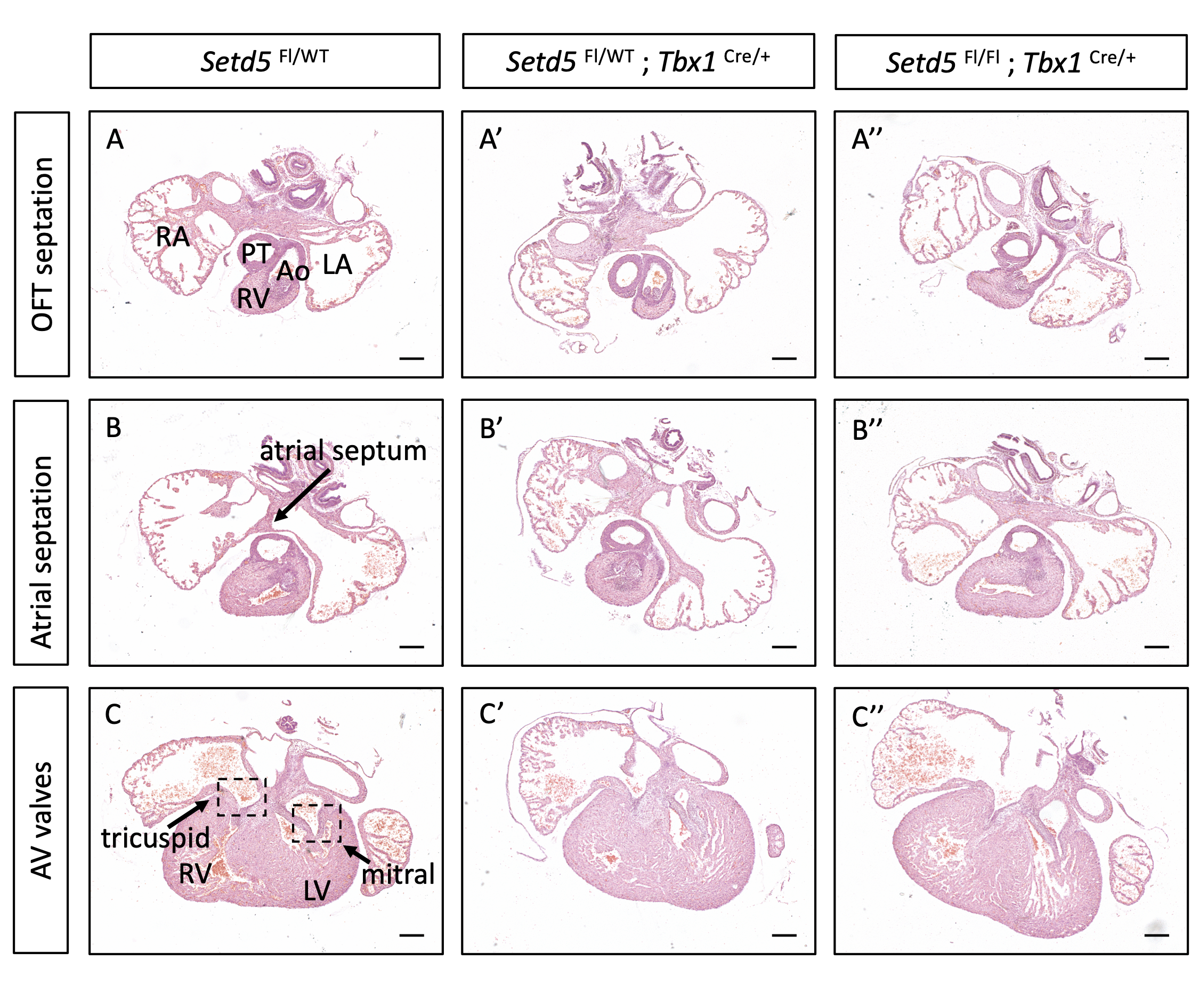

Supplement: Supplementary file 1 — Supplementary Figure 1 Transverse sections of hearts of Tbx1‐cKO (Setd5 Fl/Fl ; Tbx1 Cre/+ ) at E15.5. Panels A – A" show correct outflow tract (OFT) septation across all genotypes. The atrial septum (B – B″) and AV valves (C – C″) were intact across all genotypes Abbreviations: aorta (Ao), left atrium (LA), left ventricle (LV), pulmonary trunk (PT), right atrium (RA), right ventricle (RV). Scale bars = 200 μm. [file DVG-59-e23421-s003.tiff]

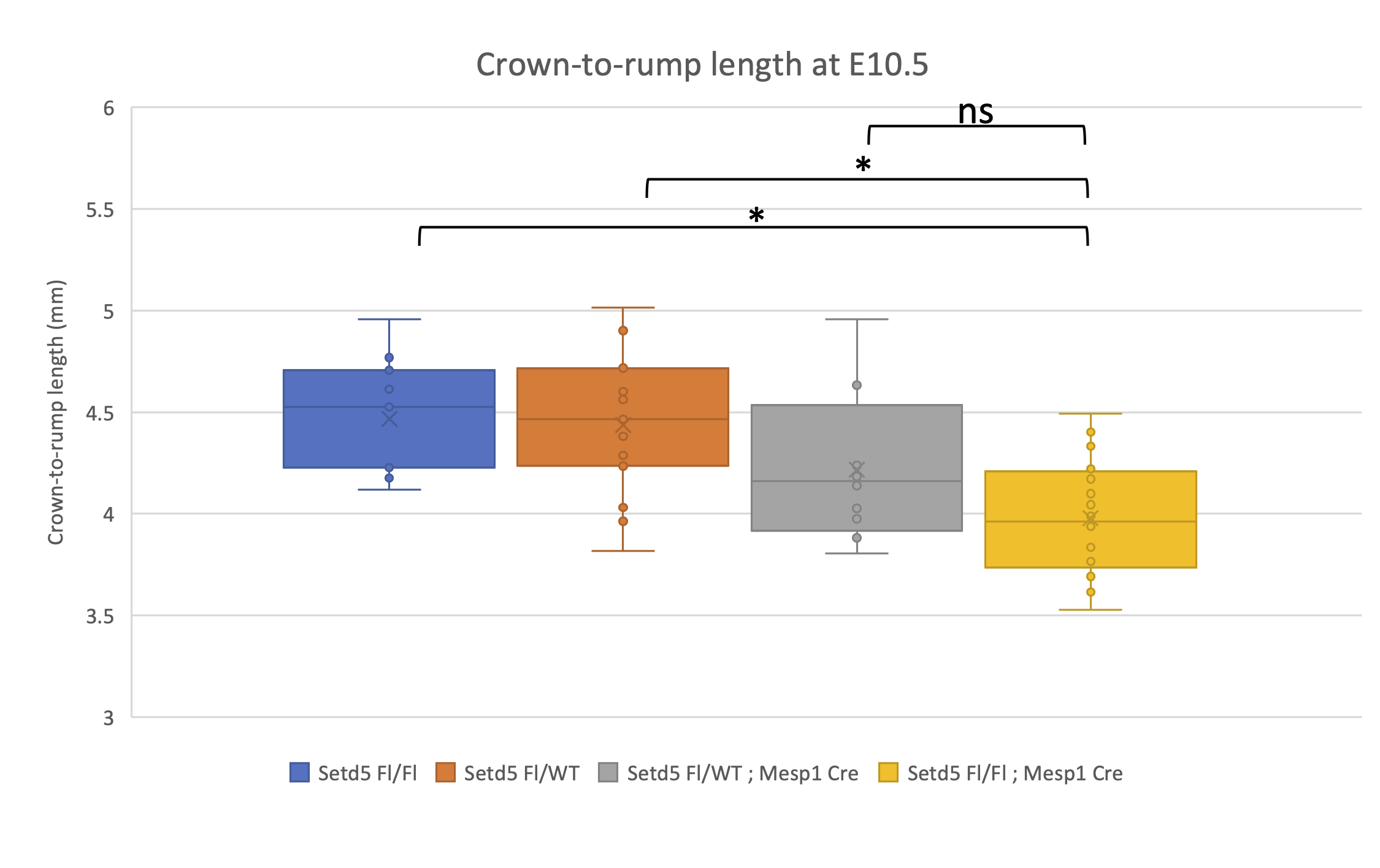

Supplement: Supplementary file 2 — Supplementary Figure 2 Box plot showing the crown‐to‐rump length in mm at E10.5. Setd5 Fl/Fl; Mesp1 Cre/+ cKO embryos are significantly smaller than control embryos (Setd5 Fl/Fl or Setd5 Fl/WT, p < .05, one‐way ANOVA). Error bars present the standard error of mean. [file DVG-59-e23421-s001.tiff]

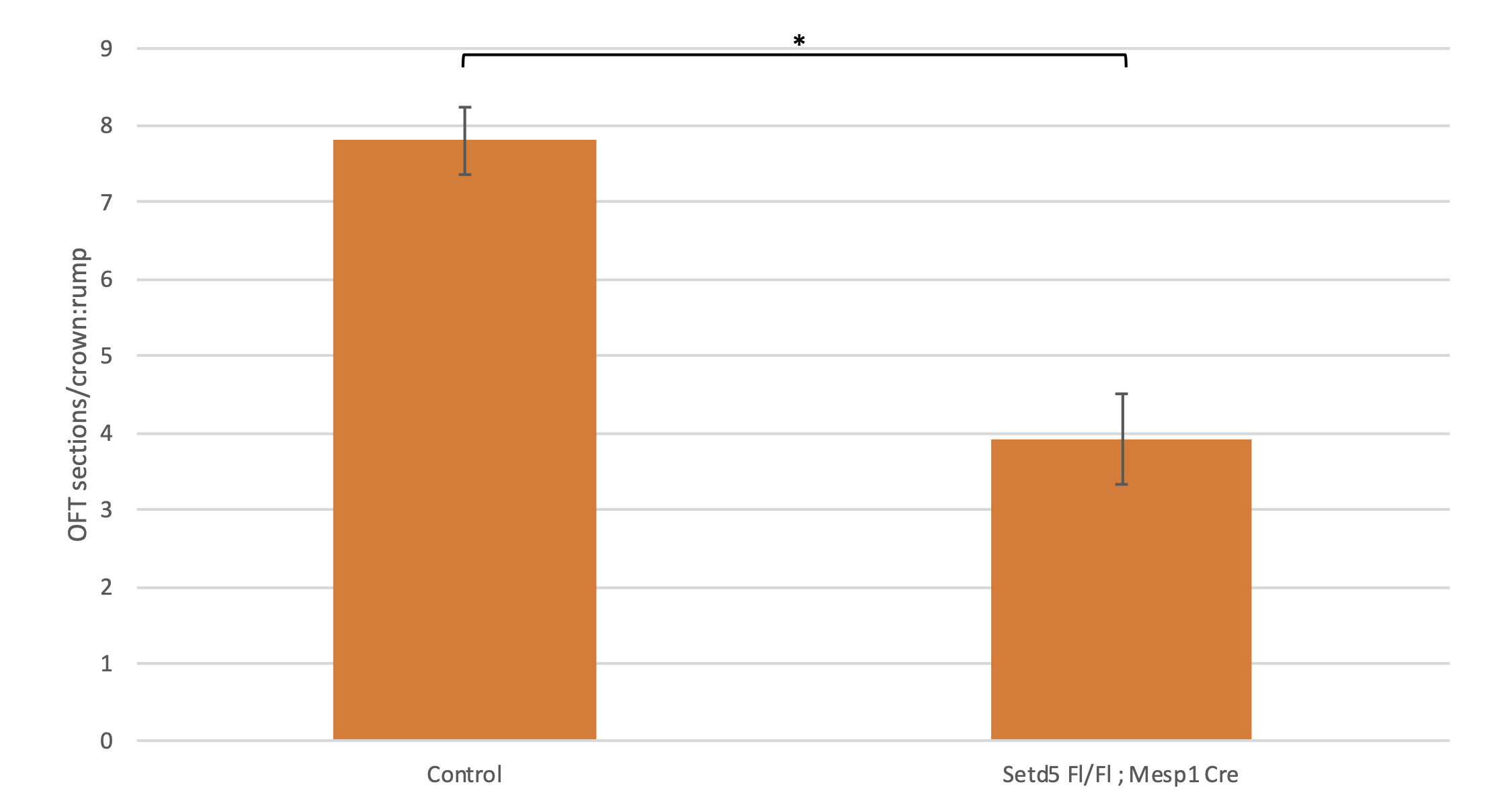

Supplement: Supplementary file 3 — Supplementary Figure 3 Graph showing the OFT length of control embryos (Setd5 Fl/Fl or Setd5 Fl/WT ) and cKO embryos (Setd5 Fl/Fl ; Mesp1 Cre/+ ) The OFT length is presented as a ratio of the number of H&E paraffin sections containing the OFT, to the crown‐to‐rump length. An unpaired student's t test showed that cKO embryos had a shorter OFT than control embryos (*p < .05). Error bars present the standard error of mean. [file DVG-59-e23421-s002.tiff]
