# Supplementary material for: Setd5 is required in cardiopharyngeal mesoderm for heart development and its haploinsufficiency is associated with outflow tract defects in mouse
Source: Genesis. 2021 May 29;59(7-8):e23421. doi: 10.1002/dvg.23421 (PMC8564859; doi:10.1002/dvg.23421)
Supplement: Supplementary file 4 — Supplementary Table 1 Observed and expected number of embryos at E14.5 resulting Setd5 +/− x Tbx1 lacZ/+ cross. Expected numbers are based on Mendelian ratios, rounded to the nearest whole number. There was no statistical difference in the number of E14.5 embryos across the genotypes, based on Chi‐squared analysis, p > .05. Supplementary Table 2: Observed and expected number of embryos at E10.5 resulting Setd5 Fl/Fl x Setd5 Fl/WT ; Mesp1 Cre/+ cross. Expected numbers are based on Mendelian ratios, rounded to the nearest whole number. There was no statistical difference in the number of E10.5 embryos across the genotypes, based on Chi‐squared analysis, p > .05. [file DVG-59-e23421-s004.docx]

**Supplementary tables**

**Supplementary table 1: Observed and expected number of embryos at E14.5 resulting *Setd5* ^+/-^ x *Tbx1* ^lacZ/+^ cross.**

Expected numbers are based on Mendelian ratios, rounded to the nearest whole number. There was no statistical difference in the number of E14.5 embryos across the genotypes, based on Chi-squared analysis, p > 0.05.

| Genotype | Observed | Expected |
| --- | --- | --- |
| WT | 15 | 16 |
| *Setd5* ^+/-^ | 15 | 16 |
| *Tbx1* ^lacZ/+^ | 18 | 16 |
| *Setd5* ^+/-^ ; *Tbx1* ^lacZ/+^ | 15 | 16 |

**Supplementary table 2: Observed and expected number of embryos at E10.5 resulting *Setd5* ^Fl/Fl^ x *Setd5*  ^Fl/WT^ ; *Mesp1* ^Cre/+^ cross.**

Expected numbers are based on Mendelian ratios, rounded to the nearest whole number. There was no statistical difference in the number of E10.5 embryos across the genotypes, based on Chi-squared analysis, p > 0.05.

| Genotype | Observed | Expected |
| --- | --- | --- |
| *Setd5* ^Fl/Fl^ | 18 | 20 |
| *Setd5* ^Fl/WT^ | 23 | 20 |
| *Setd5* ^Fl/WT^ ; *Mesp1* ^Cre/+^ | 18 | 20 |
| *Setd5* ^Fl/Fl^ ; *Mesp1* ^Cre/+^ | 20 | 20 |
